# Instance maps as an organising concept for complex experimental workflows as demonstrated for (nano)material safety research

**DOI:** 10.3762/bjnano.16.7

**Published:** 2025-01-22

**Authors:** Benjamin Punz, Maja Brajnik, Joh Dokler, Jaleesia D Amos, Litty Johnson, Katie Reilly, Anastasios G Papadiamantis, Amaia Green Etxabe, Lee Walker, Diego S T Martinez, Steffi Friedrichs, Klaus M Weltring, Nazende Günday-Türeli, Claus Svendsen, Christine Ogilvie Hendren, Mark R Wiesner, Martin Himly, Iseult Lynch, Thomas E Exner

**Affiliations:** 1 Department of Biosciences & Medical Biology, Paris Lodron University of Salzburg, Hellbrunnerstrasse 34, 5020 Salzburg, Austria; 10 Department of Geological and Environmental Sciences, Appalachian State University, Boone, USA; 11 Seven Past Nine GmbH, Rebacker 68, 79650 Schopfheim, Germany; 2 Seven Past Nine d.o.o., Hribljane 10, 1380 Cerknica, Slovenia; 3 Center for the Environmental Implications of Nano Technology (CEINT), Civil & Environmental Engineering, Duke University, Durham, North Carolina, 2770y8, USA; 4 School of Geography, Earth and Environmental Sciences, University of Birmingham, Edgbaston, B15 2TT Birmingham, United Kingdom; 5 UK Centre for Ecology and Hydrology, Pollution, Wallingford, Oxfordshire, United Kingdom; 6 Brazilian Nanotechnology National Laboratory (LNNano), Brazilian Center for Research in Energy and Materials (CNPEM), Campinas, Sao Paulo, Brazil; 7 AcumenIST SRL, Etterbeek, Belgium; 8 Gesellschaft für Bioanalytik Münster, Mendelstraße 17, 48149 Münster, Germany; 9 MyBiotech GmbH, Industriestrasse 1B, 66802 Überherrn, Germany

**Keywords:** data collection and quality control, data provenance, experimental workflow visualisation, FAIR, nanomaterial life cycle stages, study design

## Abstract

Nanosafety assessment, which seeks to evaluate the risks from exposure to nanoscale materials, spans materials synthesis and characterisation, exposure science, toxicology, and computational approaches, resulting in complex experimental workflows and diverse data types. Managing the data flows, with a focus on provenance (who generated the data and for what purpose) and quality (how was the data generated, using which protocol with which controls), as part of good research output management, is necessary to maximise the reuse potential and value of the data. Instance maps have been developed and evolved to visualise experimental nanosafety workflows and to bridge the gap between the theoretical principles of FAIR (Findable, Accessible, Interoperable and Re-usable) data and the everyday practice of experimental researchers. Instance maps are most effective when applied at the study design stage to associate the workflow with the nanomaterials, environmental conditions, method descriptions, protocols, biological and computational models to be used, and the data flows arising from study execution. Application of the InstanceMaps tool (described herein) to research workflows of increasing complexity is presented to demonstrate its utility, starting from (i) documentation of a nanomaterial’s synthesis, functionalisation, and characterisation, over (ii) assessment of a nanomaterial’s transformations in complex media, (iii) description of the culturing of ecotoxicity model organisms *Daphnia magna* and their use in standardised tests for nanomaterials ecotoxicity assessment, and (iv) visualisation of complex workflows in human immunotoxicity assessment using cell lines and primary cellular models, to (v) the use of the instance map approach for the coordination of materials and data flows in complex multipartner collaborative projects and for the demonstration of case studies. Finally, areas for future development of the instance map approach and the tool are highlighted.

## Introduction

The manipulation of matter at the nanoscale and the emergence of nanoscale materials, whose properties can be tailored by changing their size, shape, surface chemistry, and functionality, have led to the designation of nanomaterials as a key enabling technology and to their subsequent inclusion in the broader categorisation of advanced materials [1–2]. Applications of nanomaterials derive in many cases from their high surface reactivity, which results from their small size and large surface area. They include applications in catalysis [3–4] (e.g., as catalytic converters in engines and for energy capture and storage) and as sensors [5–6] (e.g., for bioremediation and environmental monitoring). In medicine [7–8] and agriculture [9–10], loading of nanomaterials with active ingredients and targeting the materials to key sites for action are enabled through surface functionalisation and the small size of nanomaterials, which allows them to access all areas. An important consequence of the reactive surface area of nanomaterials is the instantaneous interaction with their surroundings through formation of an acquired environmental or biomolecule corona [11–12] and/or via physical or chemical transformations that can occur at any of the nanomaterials’ life cycle stages [13–14].

The ability of engineered nanomaterials to change characteristics based on the properties of their environment presents a unique challenge for evaluating their potential environmental and human risks [15–16]. This “context dependence” of many nanomaterials’ properties requires distinction between extrinsic nanomaterial properties, which can change as the surroundings change (such as zeta potential, which depends on the pH value and ionic strength of the surrounding medium [17]), and intrinsic nanomaterial properties, which are not affected by the surroundings (such as bandgap and structural arrangement) [18]. This tendency of nanomaterials to change with their surroundings, or even with time during storage [19], suggests that the time between synthesis and initial characterisation and/or toxicity analysis, as well as changes in conditions of the surrounding medium, are important to document, although they are not routinely reported in the literature [20]. Baer et al. suggested that the essential history of a set of particles can be identified as provenance information that tells the origin of a batch of nanoobjects along with information related to handling and any changes that may have taken place since it was originated [21]. This would be useful in decreasing the extent of particle variability and the lack of reproducibility observed by many researchers.

Efforts to capture and document batch-to-batch variability of nanomaterials’ synthesis routes were made in the QualityNano project [22]. Also, a uniform description system for nanomaterials is to be established to describe nanomaterials (batches) uniquely and to determine when two (batches of) nanomaterials are equivalent to whatever degree specified [23]. Given the fact that nanomaterials’ similarity can only be verified through extensive physicochemical characterisation, which is often done in parallel to toxicity testing, a work-around solution was proposed, whereby projects could assign a unique identifier to their batches of nanomaterials via the European Registry of Nanomaterials [24] and add the characterisation data later, thus enabling batch similarity to be assessed by users wishing to integrate datasets. However, it is not clear whether characterisation data is added in practice, or whether any of the approaches suggested to date have been applied in a practical sense by the nanosafety research community. This could in part be due to the breadth of the nanosafety research domain; often, the researchers who produced or characterised the nanomaterials are different from those undertaking the different steps of exposure or hazard assessment. Indeed, this effect of specialisation was observed in studies of nanomaterials’ protein coronas, where the documentation of the nanomaterials’ dispersion and corona formation steps was very complete, but the description of the protein isolation and informatics steps was much less complete. This gap in documentation was attributed to the fact that the omics analyses are often performed by core facilities, and nanomaterials researchers do not know exactly what needs to be documented about these steps to enable the study to be reproduced [25].

Another frequently encountered challenge is the misconception that a statement regarding the use of a standard test guideline or guidance document is sufficient as metadata about a nanomaterial’s toxicity study to enable reuse of the resulting data. Notably these standard tests, as developed by the Organisation for Economic Cooperation and Development (OECD) are usually quite broad, as they are globally agreed upon. Thus, they allow users some flexibility in terms of medium, soil, dispersion approach and so forth, meaning that detailed documentation of each step is still required to allow others to reuse the data with confidence. This is especially important for nanomaterials, given that the test guidelines originally developed for soluble chemicals are currently being revised for the use with nanomaterials [26].

### Development of the instance map concept

The complexity and transformability of nanomaterials also has consequences for the databases used to organise and store nanomaterial characterisation and (eco)toxicity data. Databases needed to adapt to the nature of the data they were required to store. One innovative approach, taken by the NanoInformatics Knowledge Commons (NIKC) database [27], was to introduce the concept of the “nanomaterial instance” to capture the transformations that nanoscaled materials undergo in environmental and biological compartments as a visual representation to guide the data curation process [28], that is, to highlight where changes to the nanomaterial may have occurred and, thus, where additional characterisation information would be needed. Instances were designed to capture the necessary metadata needed to describe a material and its surrounding medium in mesocosm experiments while keeping the sequence of transformations intact (e.g., a material deposited in soil resulting in the material’s uptake by surrounding plants, which are then eaten by insects). Material transformations are tracked through connected instances. As originally conceived, the nanomaterial instances were used to systematically retrofit experimental data from published literature describing nanomaterials mesocosm studies in order to capture the nanomaterial transformations in a manner that sufficiently includes surrounding medium characteristics, thus representing both intrinsic and extrinsic properties of the studied material [20]. Mesocosm studies are generally complexly layered with multiple assays and characterisation methods occurring sequentially or concurrently, often within a larger encompassing study in order to gain a more complete understanding of nanomaterial behaviour. The NIKC curation team was tasked with translating these experimental studies into nanomaterial instances and identifying important metadata associated with each instance. This was done by categorising experimental data into one of five categories, namely, instance, material, medium, property, and supplementary; a property can describe either a medium (e.g., environmental, biological, or experimental) or material, a supplementary provides a way to include visual information about a property (e.g., image or diagram), and the instance itself is the point in time when material, medium, and properties are being described together. A study could have as many instances as needed to describe each of the potential material transformations. For quality assurance and quality control (QA/QC) purposes, the curation team needed a way to compare defined instances and transformations. After many trials, the most efficient method for curation was a visualisation or map that the curators would follow during the curation process; thus, instance mapping was created. More information on the approach is available in [28].

The benefits of such a visual representation for study design to guide researchers regarding which characterisation and system metadata were needed for complete reporting of nanosafety studies emerged quickly, with researchers using instance maps independently of the NIKC for purposes beyond data curation. As a project planning extensive mesocosm studies, NanoFASE adopted the concept for their mesocosm study reporting. In collaboration with their NanoCommons data shepherd [29], the NanoFASE project adopted the instance map approach for project-wide data management to structure the data reporting of the complex mesocosm experiments; the researchers used a modified version of the NIKC file format and uploaded the data onto the NanoCommons Knowledge Base [30]. These early instance maps were drawn by hand, without tools specifically designed to create these maps. Their use as an integral part of the overall data management infrastructure emerged holistically and bottom-up and evolved based on real applications by the nanosafety research community.

### Instance maps for on-the-fly data FAIRification

Much of the potential benefit provided by instance maps arises from removing the current separation of data production from data curation, harmonisation, reporting, and FAIRification (making data Findable, Accessible, Interoperable and Reuseable). Instance maps represent an integral part of data production following an on-the-fly data management approach [31], supporting all stages of the data management life cycle [29] by allowing the easy creation of a visual draft of the experimental workflow at the study design phase and then associating this workflow with the materials, environmental conditions, method descriptions, protocols, biological and computational models used, and the data produced during the study. Indeed, this use of instance maps to inform the earliest parts of the data life cycle was a primary goal of the NIKC team in developing the approach in order to generate “premeditated interoperability” of resulting datasets and, therefore, enable broad integration of datasets across multiple groups; however, the realisation of that goal could only emerge upon adoption of the approach by other research groups. The NanoCommons project pioneered the use of instance maps for documenting study design and data capture needs as part of the data shepherding approach and developed a software tool for the creation of instance maps. The approach has now been taken up and continued in MACRAMÉ and other recently funded advanced materials projects. As demonstrated here, the use of instance maps to visualise material transformations has evolved into a powerful tool that extends beyond curation and beyond engineered nanoscale materials. Indeed, researchers have started using instance maps to aid the design and planning of experiments, as communication and instructional tools at individual and collaborative levels, and in educational settings.

This paper presents examples of such new applications of instance maps for planning, documenting, and sharing study designs and associated data and metadata. The InstanceMaps tool allows users to design workflows in a fully customised manner and to connect the nodes (instances, properties, protocols, and data) with protocols and data management tools such as electronic laboratory notebooks (ELNs), which aids interoperability! While the focus of the cases presented here is nanosafety and sustainability, the general utility and applicability of the instance map concept to describe complex experimental and computational studies in other research areas and potentially in regulatory settings and industrial development and innovation processes is also evident.

## Methodological Approach

### Definition of the instance map concept

**Figure 1:**
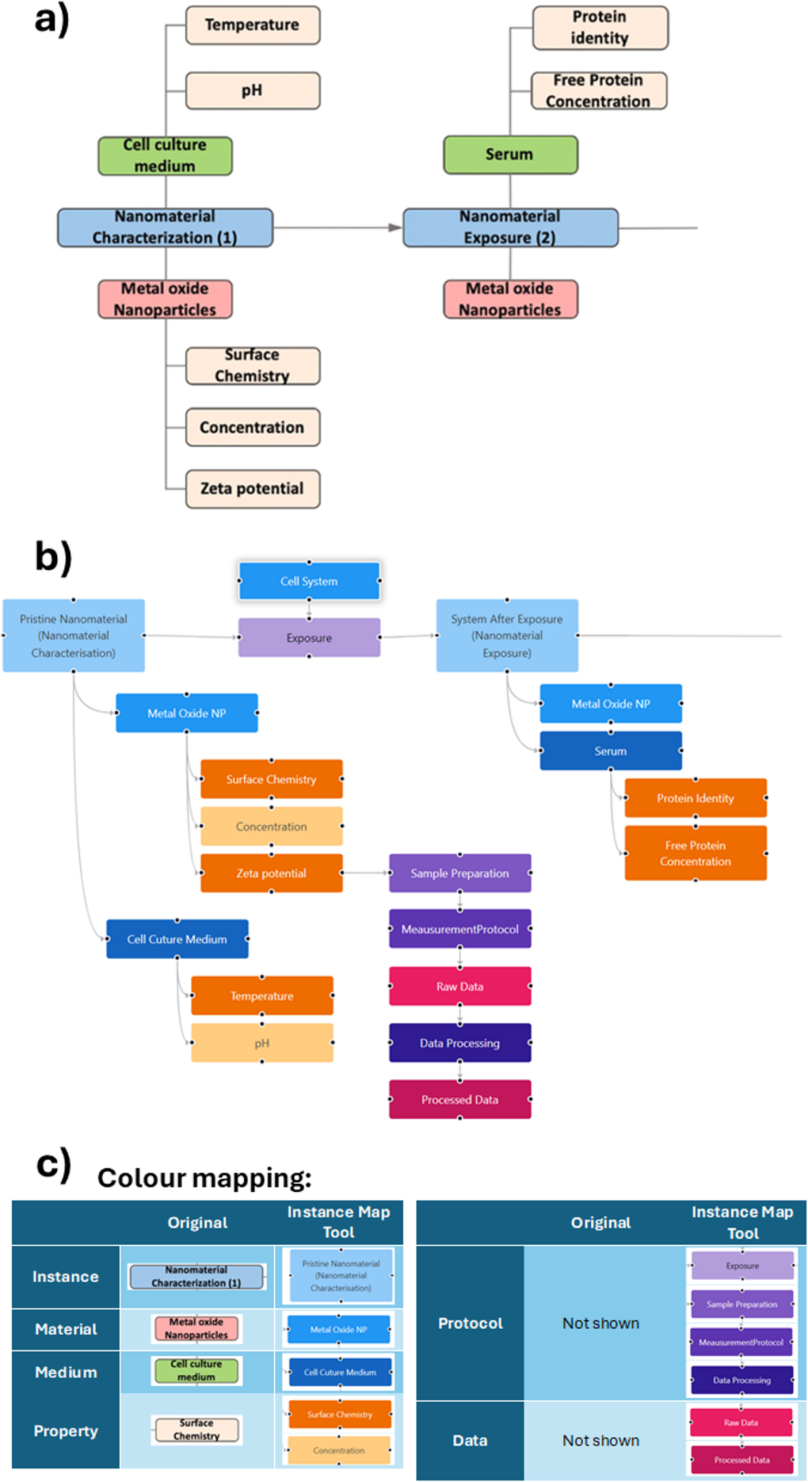
Comparison between (a) the original concept of an instance map using the original definition from NIKC, modified from Amos et al. [27] and (b) an instance map generated using the InstanceMaps tool with its extended node library. The full instance map in (b) is available at https://figshare.com/articles/software/25416040?file=51103502 for interactive inspection. (c) Comparison of the categories of instance map nodes between the original version and the InstanceMaps tool and illustration of the new features available via the InstanceMaps tool.

The original instance maps, used as organisational structure in the data curation efforts for the NIKC database [27], enabled users to visually document nanomaterial transformations while capturing the necessary metadata [28]. The experimental data is sorted into five categories, namely, instance, material, medium, property, and supplementary, to catalogue the metadata describing the nanomaterial and the exposure medium. An instance is defined as the nanomaterial in a medium at a specific moment in time. The material and medium categories are used to describe the instance. A physical or chemical change to the nanomaterial that (potentially) alters the physicochemical or biological properties of the material results in a new instance. An instance map then represents a flow chart of the nanomaterial fate, represented as a directed, often tree-like graph of nodes connected by edges, that is, arrows to show the directionality [28]. The main branch(es) is (are) formed by consecutive instances, and other branches connected to the main branch describe the material and medium at this specific point in the experiment and their properties (Figure 1).

In its original conception, the chosen categories (also called nodes) and the strict set of rules on how to place and connect the nodes was optimised for the needs of the NIKC data curators, and later for describing the mesocosm experiments of the NanoFASE project and the corresponding data curation template. The NanoCommons data shepherding services facilitated other research groups to reuse instance maps to describe their research [32–33]. These reuses also showed that a few extensions and the provision of a specialised software tool to create the maps would further facilitate and encourage the adoption for other types of experiments and new use cases, and the application of instance maps as a tool to optimise and document study design.

### The NanoCommons instance map tool

**Figure 2:**
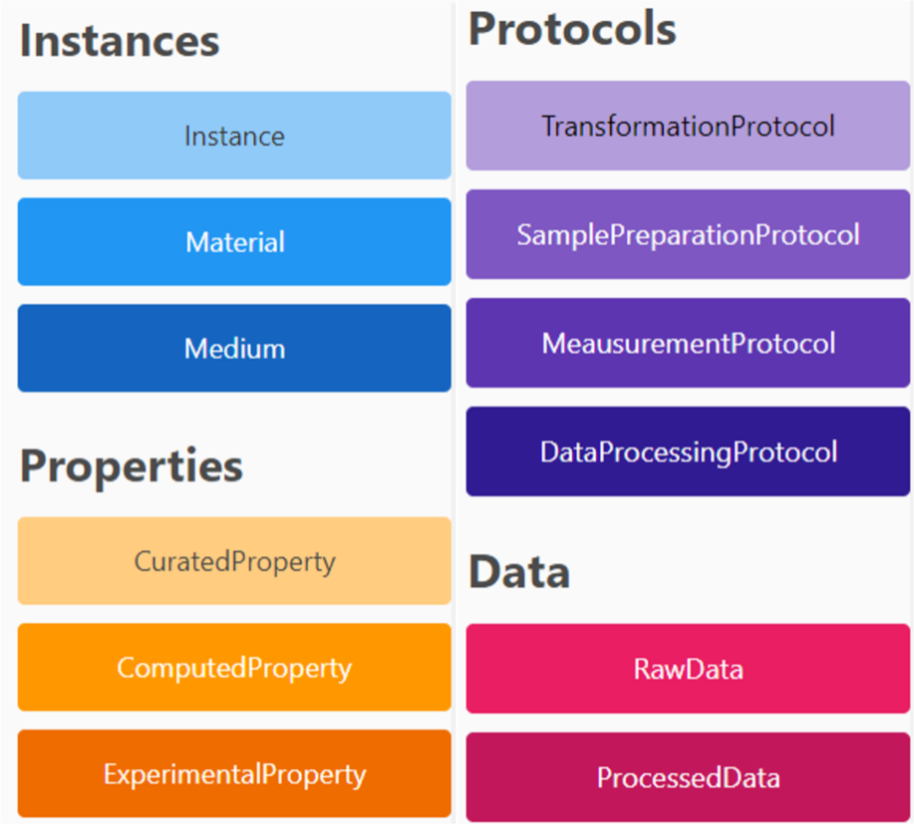
The nodes available in the InstanceMaps tool to represent a study, grouped into four categories. An instance consists of the material and its medium (surroundings). Properties can be curated (from literature) or calculated (computed) or experimentally determined. Protocols cover all steps of the workflow, including any transformations, sample dispersion and exposure, measurement steps (e.g., physicochemical characterisation, (eco)toxicity evaluation, and functional testing), and data processing such as gap-filling, data cleaning, and statistical analyses. Data is then classified as raw (coming directly from the measurement) or processed (following steps such as subtraction of medium blanks or calculation of half maximal effect concentrations).

The first extension proposed to support study design was to differentiate between different types of properties (see Figure 1 and Figure 2). In the NIKC curation efforts, all data were extracted from scientific publications; thus, there was no obvious separation in the eyes of the curator between data produced specifically within a paper (primary data) or data taken from literature or public databases (secondary data). This distinction becomes important, however, when using instance maps for complex study design workflows, where primary data can be further categorised into wet-lab and computationally produced data. To capture the complete experimental metadata, it was also seen as beneficial to be able to explicitly refer to protocols (exposure, characterisation, or toxicity) since going from one instance to the next can be a multistep process involving the application of numerous protocols and/or standard operating procedures (SOPs) of different origin. While this could be achieved by adding an instance for the resulting material state after each sub-step, these intermediate instances are not typically characterised experimentally; thus, the instances would make the maps more complex without adding much information. Explicit protocol nodes, in contrast, can be linked to the corresponding resources documenting the steps in the form of text documents, protocol repository entries, or ELN pages. For data produced in the study, a strong linkage between protocols and data using a workflow with stages for sample preparation, measurement, raw data (collection), data processing, and processed data was utilised. Putting all this together, the InstanceMaps tool supports twelve nodes, grouped into four categories, as shown in Figure 2. The node taxonomy is presented in Table 1.

**Table 1:** Taxonomy of nodes available in the InstanceMaps tool, the subjects that each node captures, and a non-exhaustive list of supporting evidence and metadata to be covered in the metadata and data files, and/or protocol descriptions associated with each node. Note that, depending on the data reporting format, some of the nodes can point to the same data file (e.g., represented as different tabs); alternatively, a full study as represented by an instance map could be stored in a single file if it supports the instance map concept.

Node	Captures	Information to be covered in the associated data file

Instance	nanomaterial (or other test compound or object of interest) in its chemical, biological, and/or product environment	listing of all components (materials and media) defining the current life cycle stage of the material/sample + bibliographic and provenance data defining the setup
Material	compositional and structural information about test object	full characterisation of material including, e.g., chemical composition, size, shape/structure, and/or NanoInChI
Medium (e.g. solvent, biological model, or product matrix)	description of the surroundings of the nanomaterial (or object of interest)	recipe of medium and/or identifier of medium and its constituents
TransformationProtocol	experimental details of changes to the nanomaterial’s/object’s surroundings that drive a change in the nanomaterials physicochemical properties	protocol document/video, SOP, and/or ELN workflow
SamplePreparationProtocol	experimental details of sample preparation, e.g., dispersion, mixing, or presentation to test organisms/environment	protocol document/video, SOP, and/or ELN workflow
MeasurementProtocol	experimental details of the measurement performed	instrument metadata, software metadata, instrument settings/input parameters, protocol document/video, SOP, and/or ELN workflow
DataProcessingProtocol	step-by-step description of the data processing	data processing pipeline, software details, statistical test details, equations utilised, and blanks/controls
CuratedProperty	description of a nanomaterial/object property extracted from a publication	bibliographic information and/or link to numeric value/data
ComputedProperty	description of a nanomaterial/object property calculated/predicted using a model or algorithm	model/algorithm name and software used to compute the property, and/or link to numeric value/data
ExperimentalProperty	description of a nanomaterial property measured experimentally	assay name, instrument metadata (if relevant), organism metadata (if relevant), metadata, and/or link to numeric value/data
RawData	data retrieved directly from observation/measurement/computation	first set of data produced by a specific experiment; what is considered “raw data” often depends on assay, context, and/or community
ProcessedData	data that has been produced following processes such as , e.g., background subtraction, normalisation, or calculation	second and any other downstream set of data generated from raw data; as for raw data, what is considered and reported here depends on assay, context, and/or community

### The refined instance map concept implemented as a web application

A first prototype of an instance map service has been developed, which speeds up the creation of the maps and allows for the linking of nodes to protocols and data sources. The tool is located at https://instance-maps.stage.sevenpastnine.com and can be accessed with a username and password (the maps of this publication can be accessed under username: SupportingInfo and password: maps-for-paper). The following functionalities are available: (i) creation and modification of instance maps and provision of basic metadata, (ii) linking of data and other research outputs to individual nodes, and (iii) sharing of instance maps with other users in the same user group, who can view the map and all associated data and (meta)data by accessing the InstanceMaps tool but cannot modify the maps.

The InstanceMaps tool was developed using a set of open-source frameworks and libraries. At the heart of the tool is ReactFlow for building node-based editors and interactive diagrams. ReactFlow is incorporated into the tool using the F#/Fable toolkit Feliz. For the backend, the Django framework is used alongside a relational PostgreSQL database to handle data storage and user management. During the NanoCommons project, a group of test users were engaged in assessing the tool’s usefulness and interface usability. Regular feedback during all phases of the development was crucial in guiding the development process with regards to defining and prioritising the requirements in terms of nodes and edges.

An instance map can be created by simply dragging and dropping items (nodes). Users can choose between the twelve different types of nodes described above, which are grouped and colour-coded for easier interpretation of map overviews. Individual nodes can be connected with edges to represent complete workflows. Data support is still limited in the current version of the tool but will be improved in the future to support the harmonised and interoperable on-the-fly data management concept envisioned in the introduction and described in [3]. Users can provide further information such as descriptions, keywords, version numbers, creation dates, licences, contributors, and references for the complete map as well as for individual nodes, as well as links (URLs or relative paths) to data files. This approach was chosen in the test phase to allow users greater flexibility with respect to the format in which their data is stored. Currently used formats for protocols and data include data serialisation formats such as JSON and YAML, notebook pages (e.g., electronic lab notebooks like SciNote, Jupyter, and Colab computation notebooks), text documents (Microsoft Word or Google Docs), spreadsheets (Microsoft Excel), and provider-specific data files. Other possibilities include images, videos, or links to public repositories. A demonstration of the tool is available at https://figshare.com/articles/software/25416040?file=51103502 along with a tutorial to support users.

## Results and Discussion

The utility of the instance map service is demonstrated on a range of experimental workflows applied in nanosafety and sustainability assessment, representing the assessment of nanomaterials or advanced materials via different endpoints and workflows. Typically, the overall experimental workflow in nanosafety assessment consists of (but is not limited to) some or all of the following steps: (i) material synthesis or procurement, (ii) further modifications (e.g., surface functionalisation), (iii) a plethora of characterisation steps by physicochemical methods, potentially also including the application of computational modelling and prediction tools, (iv) determination of diverse biological endpoints in vitro and/or in vivo, which can also consist of both experimental and computational approaches, and (v) processing of the raw data and enrichment of the processed data and its integration to support risk assessment and/or safe-by-design applications.

### Example 1: Documenting nanomaterials synthesis and provision of unique identifiers for nanomaterials

**Figure 3:**
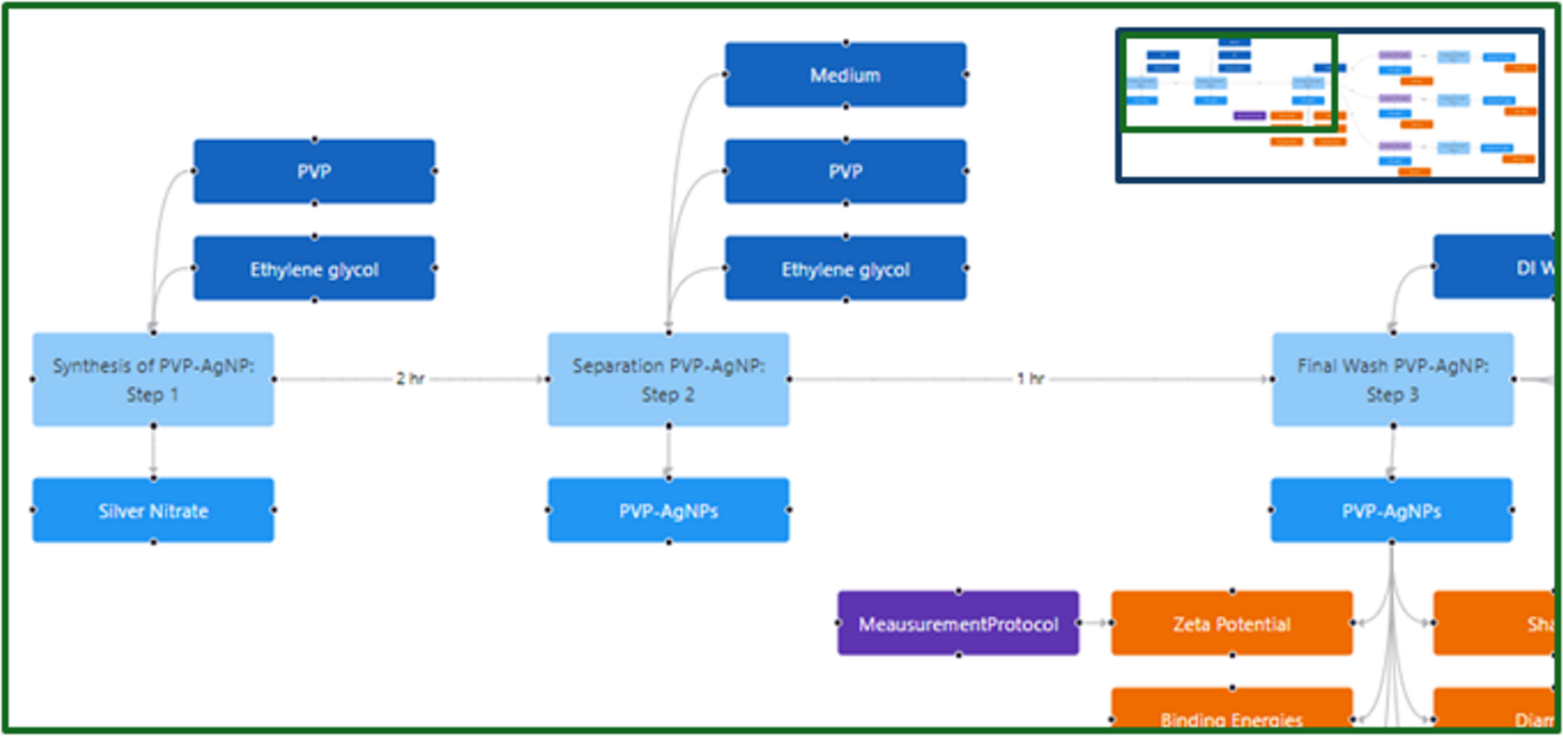
This part of an instance map shows the first steps of the synthesis protocol for the sulfidation of AgNPs originally published in [34]. The full instance map is shown as a miniature on the top right and is available at https://figshare.com/articles/software/25416040?file=51103502 for interactive inspection. The map can be divided in two phases. The first phase is the synthesis of silver nanoparticles (AgNPs) functionalised with polyvinylpyrrolidone (PVP), which occurs in the first three instances (shown as light blue nodes). Once the NPs have been synthesised, physical and chemical characterisation of the particles is performed. These characterisation endpoints can be seen in orange linked to the material in the third instance. The second phase is the sulfidation process, which can be seen in the light purple boxes (only accessible in the interactive tool). Although there are three “transformation protocols” listed, the protocol is the same except for the concentration of PVP-AgNPs used.

Instance maps were used as a tool to visualise the synthesis of different types of surface-modified nanomaterials. These maps were used to highlight how slight changes in the synthesis process can alter defining characteristics of the particle, which may drastically change particle behaviour in environmental and biological media and nanomaterial (eco)toxicity. Although multiple instance maps were created for different types of surface-modified nanomaterials, only one is presented here (Figure 3). The synthesis method illustrated was published by Levard et al. [34] and was chosen because of its thoroughly described synthesis protocol and characterisation methods. It was also chosen because the nanomaterials were later used in an extensive exposure study examining toxicity responses of organisms based on differences in the particles’ sulfidation levels [35]. We note that the same reasons underpinned its selection for the discussion of instance maps in [28].

The instance map in Figure 3 delineates all steps of the synthesis of sulfidised silver nanoparticles (AgNPs). AgNPs are synthesised with a polyvinylpyrrolidone (PVP) surface by reduction of silver nitrate in ethylene glycol with 10k PVP. The PVP-AgNPs are characterised regarding some of their physical attributes such as the particles’ shape, size, and crystalline phase. The particles are then sulfidised using different specified concentrations of the PVP-AgNPs resulting in increasing levels of sulfidation measured by the S/Ag ratio. Thus, four different NPs need to be distinguished and tracked in the subsequent toxicity experiments, leading to a need for unique identifiers for the nanomaterials.

Instance mapping is, thus, being extended to support and implement emerging standards to FAIRify nanomaterials data by creating a common naming convention. An international and interdisciplinary group is currently working on refining a standard nomenclature for nanomaterials, the “InChI for nano” or NInChI [36], based on the International Chemical Identifier (InChI). The objective is to create a notation that is readable to both humans and machines and that encompasses chemical and physical attributes of the material. As shown in the example in Figure 3, nanomaterials are often layered, often with a core and a functionalised surface, which can be engineered for specific purposes and can modulate toxicity endpoints. Ideally, a nomenclature would include details on chemical identities of the nanoparticle’s core and surface, its transformation, where it is the transformed form of the nanomaterial that is evaluated, any impurities, and physical descriptors of the material’s morphology, as well as the nature of the bonds between the surface and core. This level of detail can only be gained by understanding how the nanomaterial is synthesised, which is where instance maps will be a critical tool.

### Example 2: Monitoring nanomaterial transformation in complex environmental media

**Figure 4:**
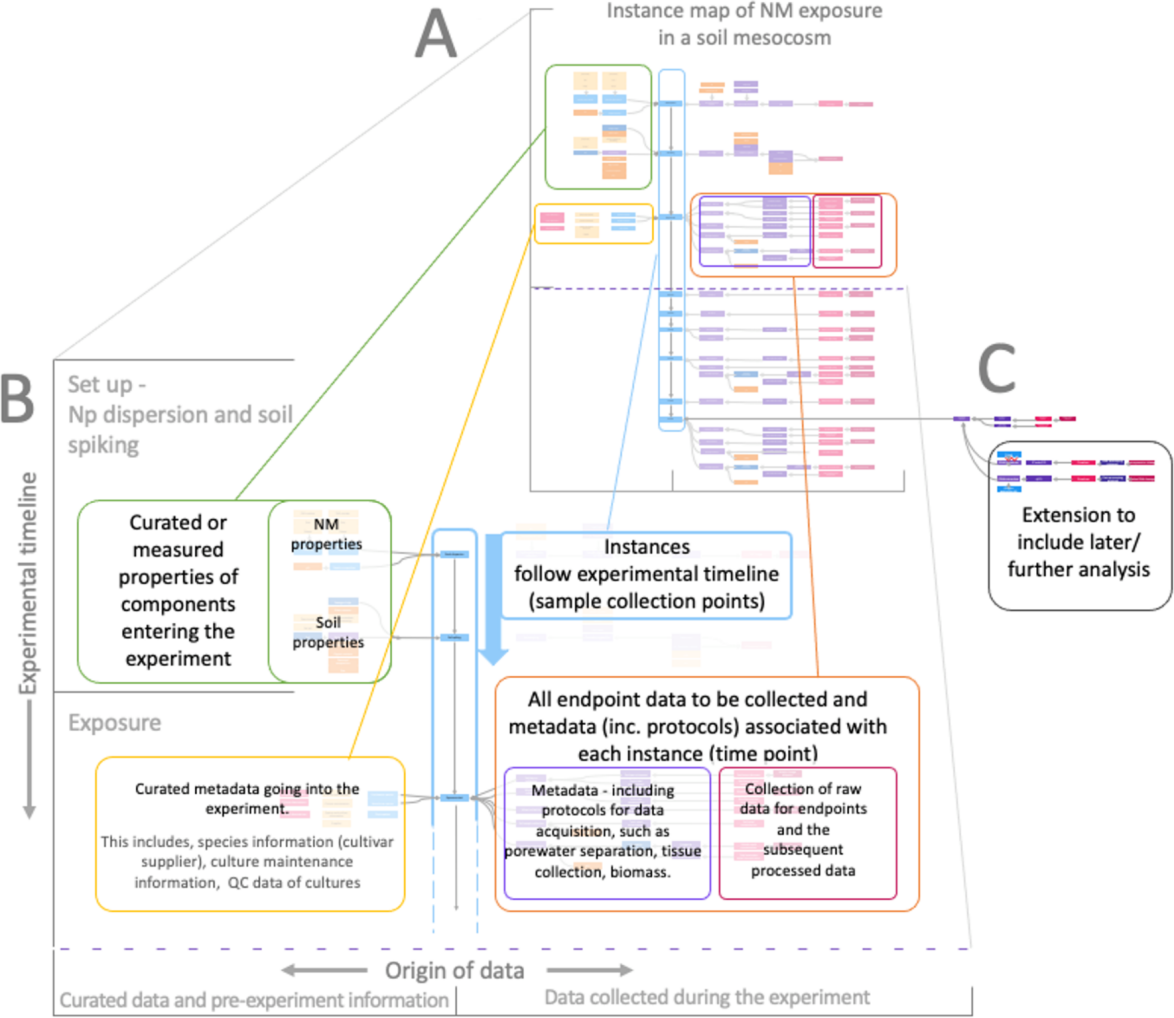
Instance map of a nanomaterial’s mesocosm experiment. (A) Representation of an instance map for a mesocosm exposure experiment. (B) An expanded map region to visualise the experiment organisation and the flow of data collection. Instances (blue boxes outlined with a blue border) are organised in time from the top of the map (blue arrow represents direction of time). Data is split on either side of the instances to distinguish its origin. To the left are the green and yellow boxes that show curated data and pre-experiment information. Curated data and pre-experiment information is further split across instances to show when it is applicable to pre- (green) or post- (yellow) exposure of organisms. To the right side of the instances is an orange box that shows all data generated from a given instance. This data is also further split into two categories. First, raw data and processed data (pink border) and, second, the methodologies and processing approaches used to derive that data (purple border). (C) Extension of a sample node to include further analysis and data points. The full instance map is available at https://figshare.com/articles/software/25416040?file=51103502 for interactive inspection.

Ecotoxicity exposures conducted in soil and mesocosm experiments are often complex with multiple parameters and endpoints (e.g., [37–40]). The diversity of data types required to monitor soil, porewater, nanomaterials, and organisms requires many sample collections and analyses; also pre-experiment data and metadata need to be collected. The complexity of the experiments is simplified by the use of instance maps, which allow for an overview of biological and chemical sampling during the mesocosm experiment. By detailing all relevant metadata and post-exposure analyses, instance maps visualise the flow of data collection and methodologies, including the biological culture information and chemical pre- and post-exposure data (Figure 4A).

The instances in the example in Figure 4 follow the timeline of exposure; at each instance, the nodes depict the data pertaining to that particular instance. Instances at the top of the map (see Figure 4, “Set up – nanomaterial dispersion and soil spiking”) occur before the exposure of organisms and include the nanomaterial dispersion and their addition to soil, followed by instances detailing the addition of organisms and then the timepoints of sample collection. Data was organised left to right to visualise the distinction between curated data and/or any pre-experiment information and data generated by the experiment itself (Figure 4B). The data and processes are visualised as nodes attached to each instance. On the left side of this example are nodes relating to the components prior to their addition to the experiment, that is, information on the pristine nanomaterial, medium and soil, suppliers, batch numbers, CAS numbers, pH, and any other pre-processing steps before the addition into the experiment (mesocosm). For the first exposure instance, also species information such as cultivars, suppliers, culture maintenance information, and QC of organisms entering the experiment are included.

On the right side of the close-up (Figure 4B), an organised display of any data generated by the experiment itself is shown. This is split into raw and processed data, as well as the processes of their collection. The pink section nodes represent the raw data sets collected, such as the pH value of soil after the addition of the nanomaterial, organism biomass data, metal concentrations in organism tissues, and any processed data derived from this raw data, such as EC50s or metal bioaccumulation rates into organisms. Information regarding the protocols and methods used for data collection and how samples were processed is also attached to the data. For example, soil porewater separation protocols, needed to help generate porewater metal concentration data, and all tissue sample collection processes are available.

The level of overview provided by instance maps greatly benefits the complex, multi-endpoint experiments common to ecotoxicology, ensuring metadata collection and optimal experimental design, and informing sample processing schedules and data management plans. The flexibility of the instance map system means that maps can be extended to include any further branching to processes, such as adding any later analysis of collected samples by extending a branch for that sample, for example, transcriptomic analysis on exposed organisms by real-time polymerase chain reaction (Figure 4C).

### Example 3: Linking assay QA/QC with SOPs for running cultures of biological organisms and standardised ecotoxicity testing

Keeping records of the normal organism behaviour in individual labs is vital for regulatory testing, but it is not something that is formalised in most academic laboratories. Thus, instance maps can also be used to build awareness of the pre-experiment steps and the importance of documenting these as they form part of the provenance and QA/QC metadata that underpin regulatory testing. This data supports demonstration of the trustworthiness of (hazard) data to others who may wish to reuse the data (e.g., in modelling or as part of a risk assessment).

**Figure 5:**
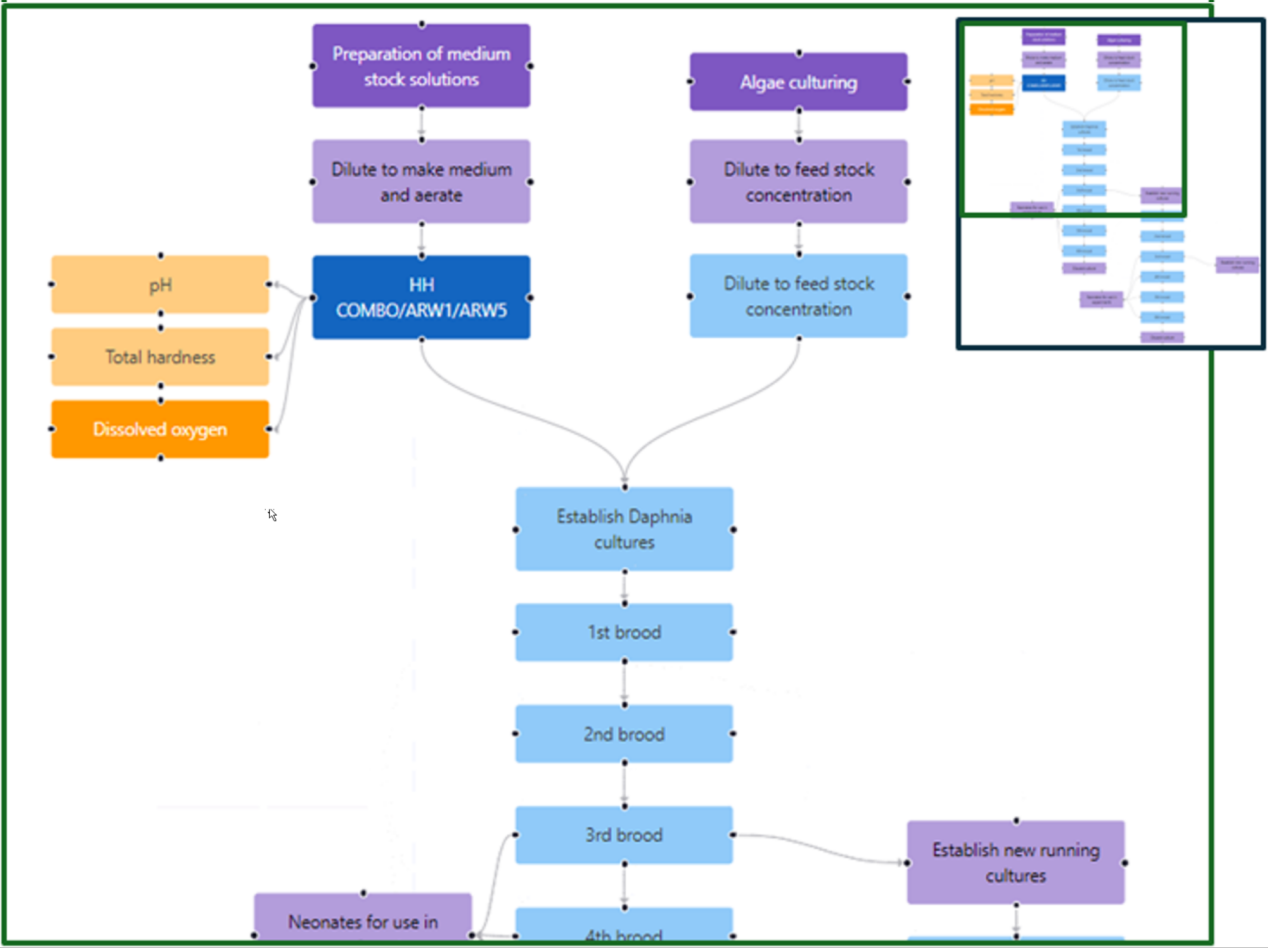
Instance map visualising the steps in maintaining continuous *D. magna* cultures. Daphnia typically produce brood from about ten days of age and roughly every three days thereafter, with the third to seventh broods being the most genetically stable and, thus, suitable for ecotoxicity experiments. Tracking of the number of offspring per brood is one of the essential QC measures to record, using the template shown in Table 2. Details such as organism species, strain, and culturing conditions (temperature, pH, dissolved oxygen, light/dark cycle) can be captured here as well as the specifics of, for example, the medium and the culturing vessels. The full instance map is available at https://figshare.com/articles/software/25416040?file=51103502 for interactive inspection.

The model organism *Daphnia magna* is cultured in a high hardness medium, which is aerated for a minimum of 8 h prior to use in culturing; the dissolved oxygen content is measured every 2–3 days to ensure it stays within the acceptable range. The pH value of the medium is also measured and moderated to within the defined parameters for the specific medium before use for the ongoing culturing of daphnia. The running cultures are typically in large (1 L) beakers with 900 mL medium and can contain 10–15 adults, with the medium being refreshed three times per week. All cultures are fed the same daily algal ration of *Chlorella vulgaris* (7.5 mg C days 0–7, 11.25 mg C days 7 onwards, with double rations on Fridays to cover the weekend) and are kept in a 20 °C laboratory under a 16:8 hours light/dark cycle. The steps involved in maintaining the daphnia and the algae on which they feed are shown in the instance map of Figure 5. Third-brood daphniids are used for all ecotoxicity experiments (i.e., acute and chronic toxicity testing) to ensure optimum genetic health of future cultures.

**Table 2:** A simple data capture template for monitoring the health and performance of running daphnia cultures, wherein the amount of food and dates of medium changes are reported along with the numbers of offspring measured per culture jar. Long term tracking of culture performance allows for confidence in data generated in regulatory testing using standardised assays, as any deviations from normal behaviour can be confirmed as being from the exposure rather than from any anomalies in how the test was performed.

Date	Day of culture	Culture (number in jar)						Offspring						Food (mL)	Medium change	Comments

		1	2	3	4	5	6	1	2	3	4	5	6			

02/01/24	1	12	12	12	12	12	12							0.75	√	
03/01/24	2	12	12	12	12	12	12							0.5		
04/01/24	3	12	12	12	12	12	12							0.75		
05/01/24	4	12	11	12	12	12	12							1		
06/01/24	5	12	11	12	12	12	12							1	√	
07/01/24	6	12	11	12	12	12	12							2		
08/01/24	7	12	11	12	12	12	12							0		
09/01/24	8	12	11	12	12	12	12							1.5	√	
10/01/24	9	12	11	12	12	12	12							1.5		
11/01/24	10	12	11	12	12	12	12							1.5		eggs in brood pouch
12/01/24	11	12	11	12	12	11	12	√	√	√		√	√	1.5	√	first brood
13/01/24	12	12	11	12	12	11	12				√			3		
14/01/24	13	12	11	12	12	11	12							0		
15/01/24	14	12	11	12	12	11	12	√	√	√		√	√	1.5	√	
16/01/24	15	12	11	12	12	11	12				√			1.5		
17/01/24	16	12	11	12	12	11	12							1.5		

**Figure 6:**
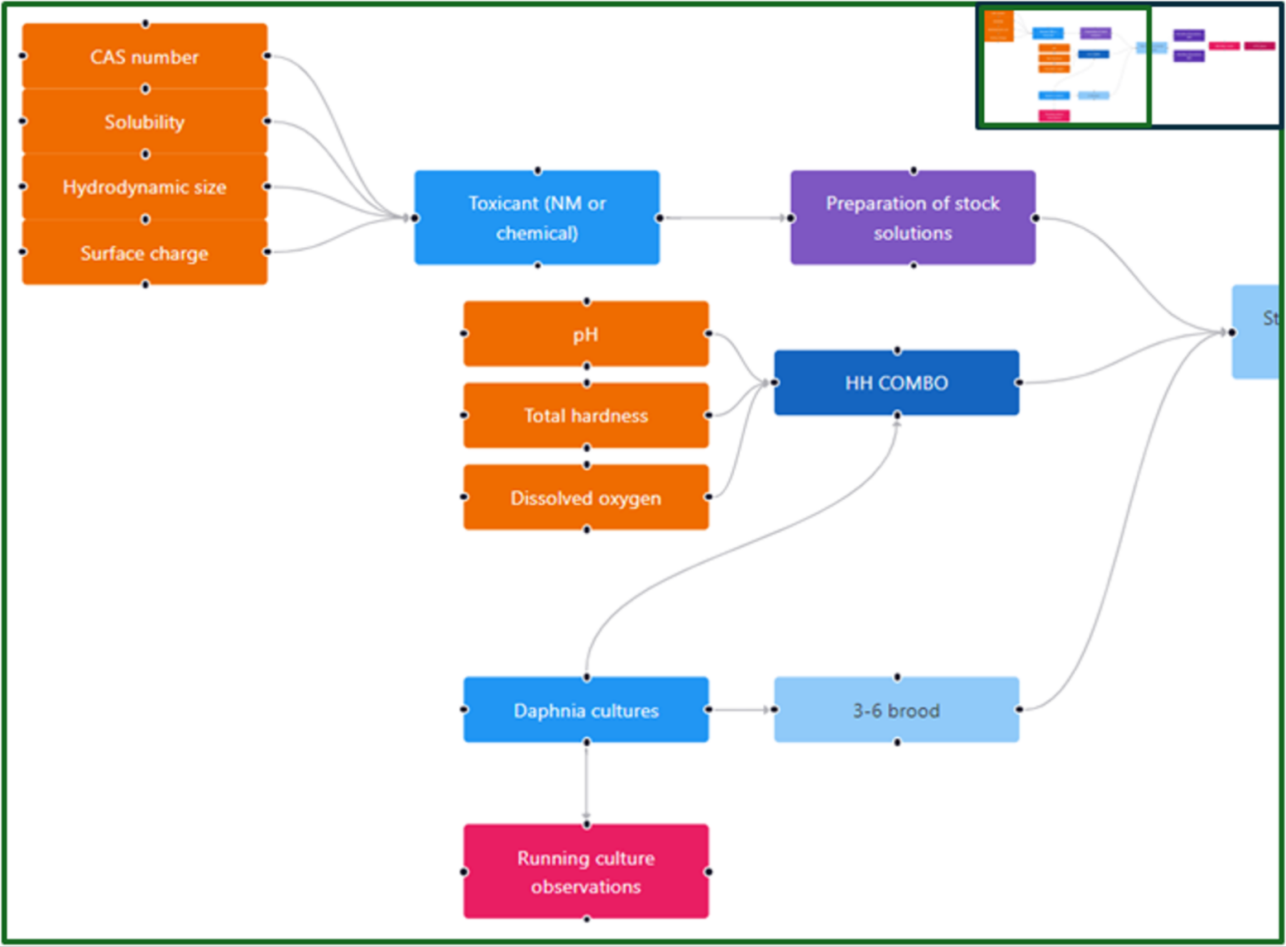
Representation of the OECD 202 “*Daphnia* sp. acute immobilisation test” guideline for acute toxicity to daphnia as an instance map. For nanomaterials, there would be an additional link from the stock solution to the range of characterisation studies needed, such as size distribution, surface charge, and stability over time. The full instance map is available at https://figshare.com/articles/software/25416040?file=51103502 for interactive inspection.

**Figure 7:**
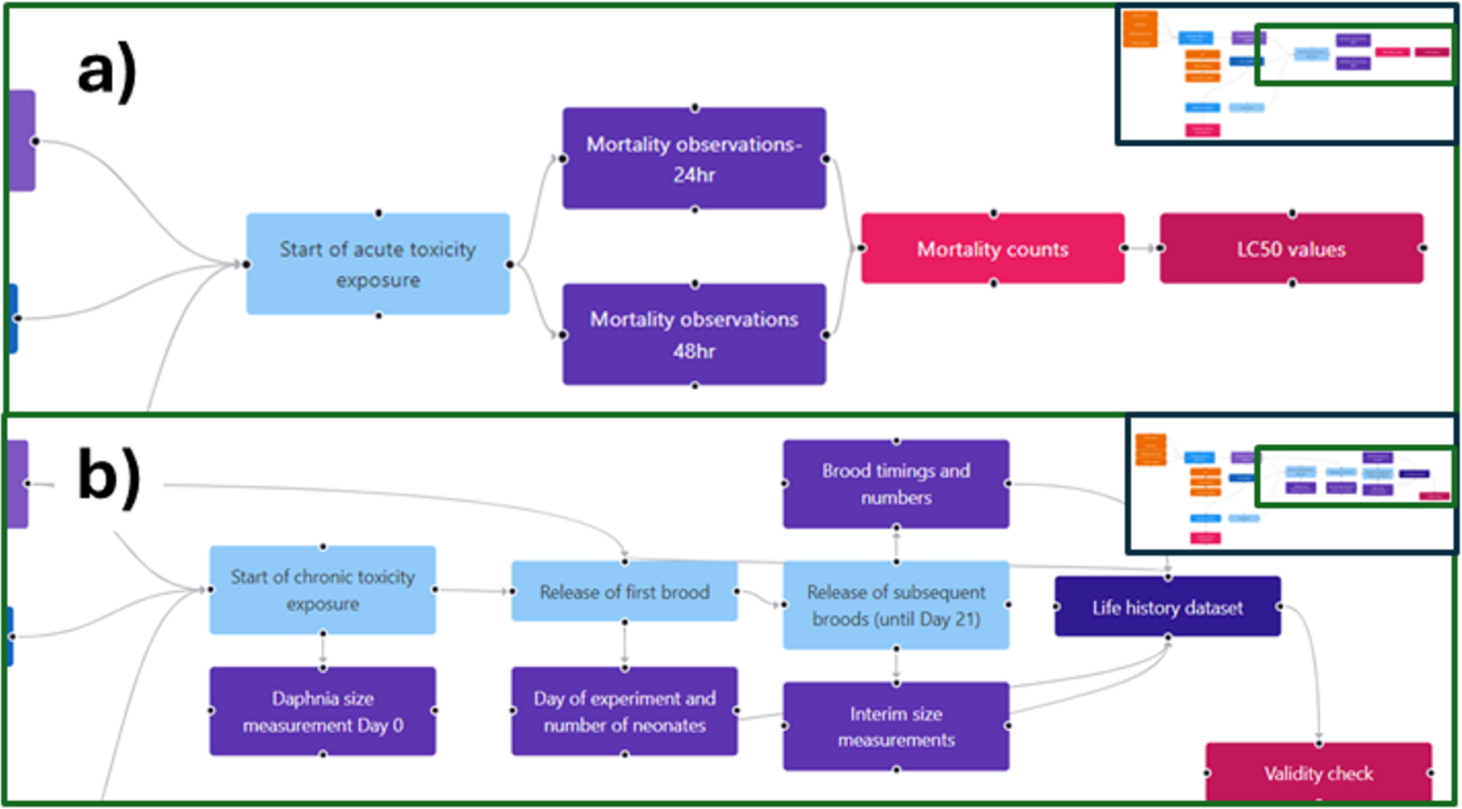
Representation of the OECD 211 “*Daphnia magna* reproduction test” guideline for reproductive (chronic) toxicity to daphnia as an instance map (b). The exposure concentration is determined from the acute dose–response curve (a), generated according to the instance map shown in Figure 6, which will be fully integrated in a next iteration of the InstanceMaps tool. The instance map is available at https://figshare.com/articles/software/25416040?file=51103502 for interactive inspection.

The steps in the acute daphnia toxicity test performed according to the OECD standard test guideline (OECD 202 “*Daphnia* sp. acute immobilisation test” [41]) have been visualised using an instance map (Figure 6). An intentional feature of the OECD test guidelines is that they leave some flexibility for the users in that they recommend a specific medium, but this is not essential (and indeed many labs use tap water or bore hole water). Thus, each lab needs to prepare its own detailed SOP that underpins the experiment. In the example shown, we have not linked to other aspects of an overall study that would be required, such as characterisation of the stock solution and assessment of the nanomaterials’ stability in the test medium. However, the beauty of the instance map approach is that this linking of experiments/experimental steps is easy. A related data capture template has been developed and is linked to the raw data node. The OECD 211 “*Daphnia magna* reproduction test“ (a reproductive assay) has also been mapped, as shown in Figure 7, noting that the concentration used in the chronic test is usually derived from the acute test (e.g., the EC30 or EC10 concentration). Thus, these instance maps can also be linked and are indeed linked to the running culture instance map of Figure 5.

In line with the QA/QC efforts presented here, initiatives are ongoing at the European level, and to a certain extent even global level, aiming, for example, at the harmonisation of nanomaterials characterisation reporting, its terminology, classification, and metadata. A standard structure containing this type of information relating to (i) materials characterisation (meta)data, termed CHADA (CHAracterisation DAta and description of a characterisation experiment), has recently been proposed [42]. Standardised or harmonised reporting formats had previously been called for, such as a listing of minimal reporting standards for biological assays studying the interactions of nanomaterials with biological materials, termed MIRIBEL [43]. The prime intention here is to improve future exchange of datasets among materials characterisation experts, to facilitate collaboration with industry end users, and to optimise the interoperability of data and, thus, enable better data reuse by modelling experts. Likewise, efforts are ongoing to harmonise the (ii) materials MOdelling DAta terminology, resulting in templates for physics-based model description, termed MODA [44], driven by the activities of the European Materials Modelling Council (EMMC), resulting in a workshop agreement of the European Committee for Standardization (CEN). Instance maps can support this effort by graphically resolving reporting documents as they enable a structural representation of the experimental (or even computational) data workflow. In the context of biological experimentation, we can link the instance maps to (iii) biological data reporting that fulfils criteria such as advocated by MIRIBEL. Analogous to the two aforementioned reporting formats, such a biological documentation could be termed BIODA, a reporting structure for BIOlogical assay DAta. Such concepts will prove useful when highly complex workflows are built and data has to be aggregated from the different criteria. For example, regarding the regulatory readiness of testing pipelines based on new approach methodologies, batteries of more than 20 assays with over 50 individual endpoints are compared and data (from different laboratories) needs to be aggregated [45]. The offspring tracking presented in Figure 5 may represent the first step towards implementation of a BIODA to allow for benchmarking and interoperability of data from different labs, similar to the CHADA and MODA concepts.

### Example 4: Showcasing complex workflows in human immunotoxicity assessment using cell lines and primary cellular models

**Figure 8:**
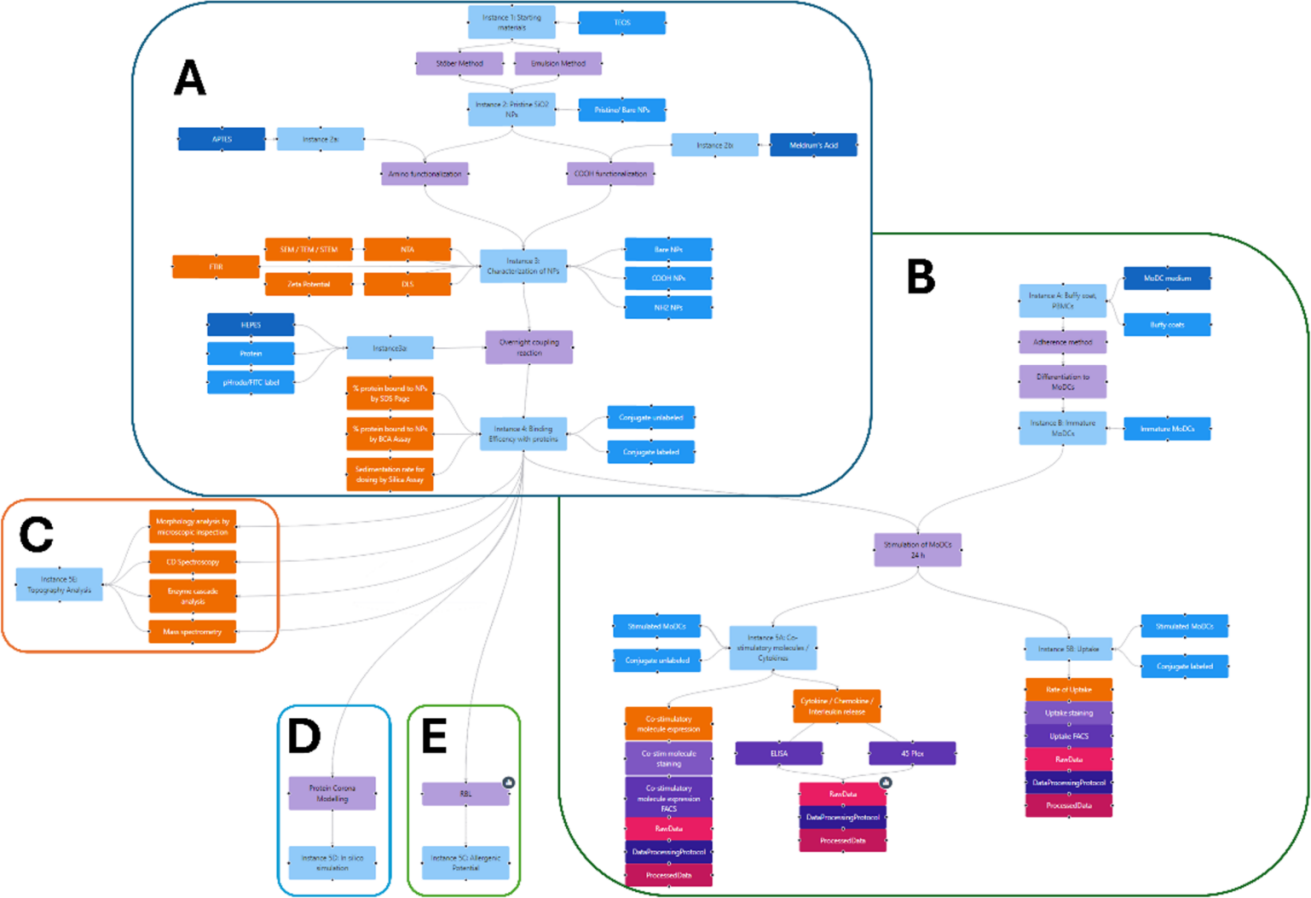
Instance map of the immunotoxicity workflow to study the bio-nano interactions of differently functionalised SiO_2_ nanomaterials with immune cells. The instance map is divided into sections A–E, based on the studies of Hasenkopf et al. [48], Mills-Goodlet et al. [49], Johnson et al. [50], and Punz et al. [51], highlighting the different approaches and routes that were taken. Section A, which serves as a baseline for all studies, mainly focuses on the nanomaterial synthesis and surface modification. The pathway towards more in-depth immunological investigations was chosen for section B, while sections C and E cover alterations in the protein binding activity, depending on the physicochemical properties provided by chemical surface functionalisation and also the observed structural alterations that occurred upon nanomaterial conjugation because of the nanotopography of the materials (mesoporous SiO_2_ nanomaterials). Section D depicts the integration of in silico predictive modelling approaches with quantitative and qualitative in vitro determination of the protein corona (epitope rearrangement). The full instance map is available at https://figshare.com/articles/software/25416040?file=51103502 for interactive inspection.

In the context of studying bio-nano interactions of silica-based nanomaterials with potential use as adjuvants in immunotherapy and of allergens as active pharmaceutical ingredients (APIs), we used the InstanceMaps tool to summarise and highlight different workflows for investigating immunotoxicity and pharmacologic efficacy endpoints. Regarding materials, the studies focused on silica (SiO_2_) nanomaterials in the size range of 50–100 nm (depending on the method used, i.e., transmission electron microscopy, nanoparticle tracking analysis, or dynamic light scattering (intensity or number distribution)) with different surface modifications, which are reported to be immunologically active in different ways but are overall considered to be safe [46]. In some studies, the impact of material surface (nanotopography) and functional modifications on API binding (molecular initiating event according to the “adverse outcome pathway” concept [47]) were investigated. In other studies, the different steps involved in specific immune reaction mechanisms (key events for beneficial vs adverse outcomes) were analysed. Figure 8 illustrates the comprehensive experimental workflow overarching several immunotoxicity studies, highlighting the different routes chosen for these studies, including synthesis, surface functionalisation, and physicochemical characterisation of the nanomaterials, the bio-nano interaction studies, and the determination of different biological/immunological endpoints.

When studying bio-nano interactions the starting point is typically the synthesis (or procurement) of the particles, which for SiO_2_ nanomaterials is either the Stöber method or the emulsion method, followed by chemical modification. Here, nanomaterial functionalisation was realised by addition of amino or carboxyl groups with shorter or longer aliphatic linkers. Alteration in the particles’ nanotopography was realised through pore formation during synthesis using cetyltrimethylammonium bromide. The non-covalent conjugations between nanomaterials and proteins were quantitatively characterised, directly by gel electrophoresis and indirectly by quantifying the amount of unbound protein in the supernatant upon several washing steps. “In Vitro Sedimentation, Diffusion and Dosimetry” studies were undertaken to determine the cell-delivered dose for all culture conditions based on the specific density and size parameters of the bio-nano conjugates [52]. Finally, comprehensive physicochemical characterisation was performed by applying a set of analyses according to the reporting standards for bio-nano interactions [43], and (meta)data were uploaded to the NanoCommons Knowledge Base [30], following the principles for data FAIRness and metadata stewardship [29]. These were the necessary baseline requirements to proceed with experiments, which are defined as section A in Figure 8.

The sections concerning the biological and immunological readouts, as well pharmacological efficacy, independently expand upon section A. The focus in Figure 8B is on mechanistic studies following uptake and presentation by professional antigen-presenting cell (APC) models using unmodified SiO_2_ nanomaterials [50] compared with differently surface-functionalised particles [51]. As a model for APCs, monocyte-derived dendritic cells were generated from human whole blood samples as a preliminary step, again building a BIODA-type of reporting structure following SOPs. Afterwards, these APCs were incubated with the materials generated in section A, and their immunologic activation profile was investigated utilising flow cytometry and enzyme-linked immunosorbent assay. Sections C and E in Figure 8 are quite similar in concept [49] and investigate the influence of nanotopography on the protein binding capacity and its impact on epitope integrity. Johnson et al. [53] reported that structural alterations of proteins bound to nanomaterials impact the antigen-processing machinery in APCs and could, thus, impact the outcome in terms of immunomodulation. Here, it should be emphasised that during immunotherapy against type-2 immune diseases, such as allergies, a shift towards regulatory T cell activation is envisioned. Finally, as depicted in section D, Hasenkopf et al. [48] tested the proteins’ individual binding efficiencies on differently functionalised SiO_2_ nanomaterials under varying conditions. They also compared artificial and real allergen mixtures by applying genuine detection assays suitable for allergenic molecules in vitro and assessed two recently developed in silico protein corona prediction tools regarding the results from experimental studies.

The aforementioned studies are complex and individually targeted to different endpoints. The InstanceMaps tool allows users to generate large and intertwined workflows referring to multiple research objectives. While a single experiment can already be depicted by an instance map, we herewith displayed their use for visualising integrated batteries of assays and depicted their applicability as a structural representation of larger collaborative research and development endeavours. Instance maps have thus proven instrumental as a tool for creating and illustrating workflows that combine several sophisticated backgrounds, allowing even less experienced users to capture the bigger picture and still perceive more detailed correlations within a larger context.

### Example 5: Using instance maps for planning and refining data and material workflows in large collaborative projects

As the last example of application and utilisation of instance maps, their use for reporting of studies with complex workflows and as a tool for study design and planning and tracking tool for materials, samples and data flows is presented. The MACRAMÉ project aims to extent the coverage and widen the applicability domains of harmonised OECD test guidelines, OECD guidance documents, and international standards (CEN, ISO) by refining existing and developing new advanced physicochemical, human, and ecotoxicity characterisation methodologies for market-relevant nanomaterials and the wider group of advanced materials [54] in their complex product matrices. Applicability, relevance, and reliability are tested in five industrial use cases. To demonstrate the instance-map-based data management approach of MACRAMÉ, the use case of antibiotics-loaded polymeric nanomaterials is showcased. These nanomaterials are used for a proof-of-concept of the treatment of antibiotic-resistant bacterial lung infections. In addition, controls are prepared for imaging purposes to verify the suitability of the MACRAMÉ approach to quantify and characterise the aerosols upon exposure of in vitro lung models. Relevant exposure points were identified and used to define the samples that need to be taken from the industrial processes and sent to the characterisation and testing partners. The combinations of advanced materials and complex matrices to be studied include all life-cycle-relevant occurrences of (i) complex product matrices, (ii) degraded complex product matrices at the product’s end of life, (iii) regulatory relevant biological matrices for human toxicity testing, (iv) environmental matrices for ecotoxicity testing, and (v) relevant forms of the different complex matrices, such as soot and char, and aerosols generated from compounding, machining, use, weathering, degradation, or incineration of products.

To achieve such a full characterisation of the materials along their complete life cycle and, at the same time, move the methods forward on their road to standardisation – all in the short time of the project – intensive collaboration and unhindered knowledge exchange between all partners is essential. Flows of material and data/information, from production to sample preparation (simulating different end-of-life scenarios) to collection of the characterisation data, need to be organised effectively in order to satisfy the information requirements of downstream experiments; also, all data needs to be integrated to perform a safety and sustainability evaluation.

**Figure 9:**
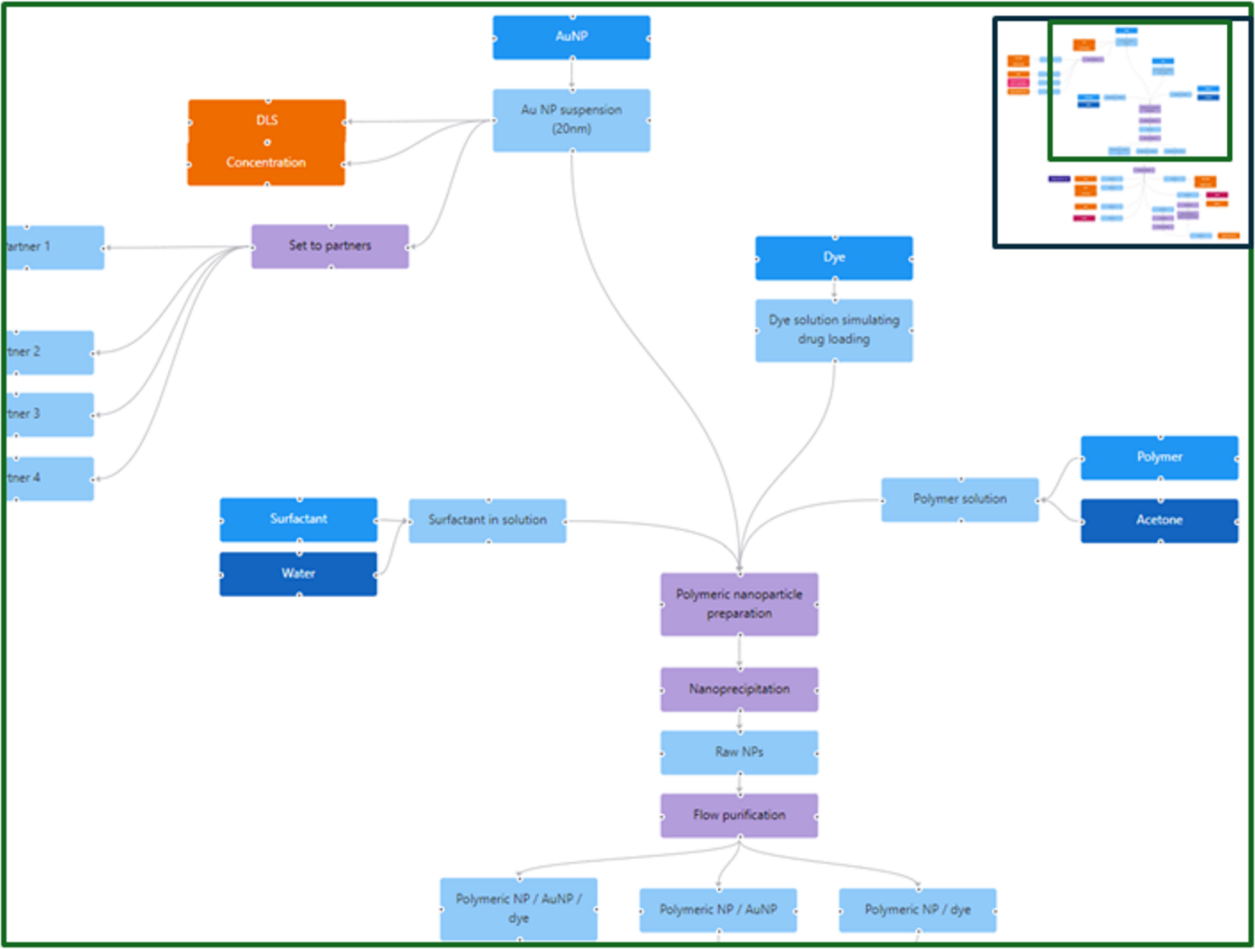
Part of the instance map depicting the planning status of the human and ecotoxicology testing for the MACRAMÉ use case of antibiotics-loaded polymeric nanomaterials. After the production of the loaded nanomaterials, they are sent to many experimental partners performing the different assays. The instance map is crucial to describe the complexity of the workflow, which includes strong cross-partner dependencies such as sample preparation by one partner and measurement by another, which must be completed within a specific timeframe. The full instance map is available at https://figshare.com/articles/software/25416040?file=51103502 for inspection.

The InstanceMaps tool was used to visually map all exposure points, the characterisation methods applied to these points, and the workflows needed to create the materials and the life cycle samples and to execute the experiments. This ensures that all information required to perform the safety and life cycle assessment is collected, with all steps documented as part of the planning status (see Figure 9).

**Figure 10:**
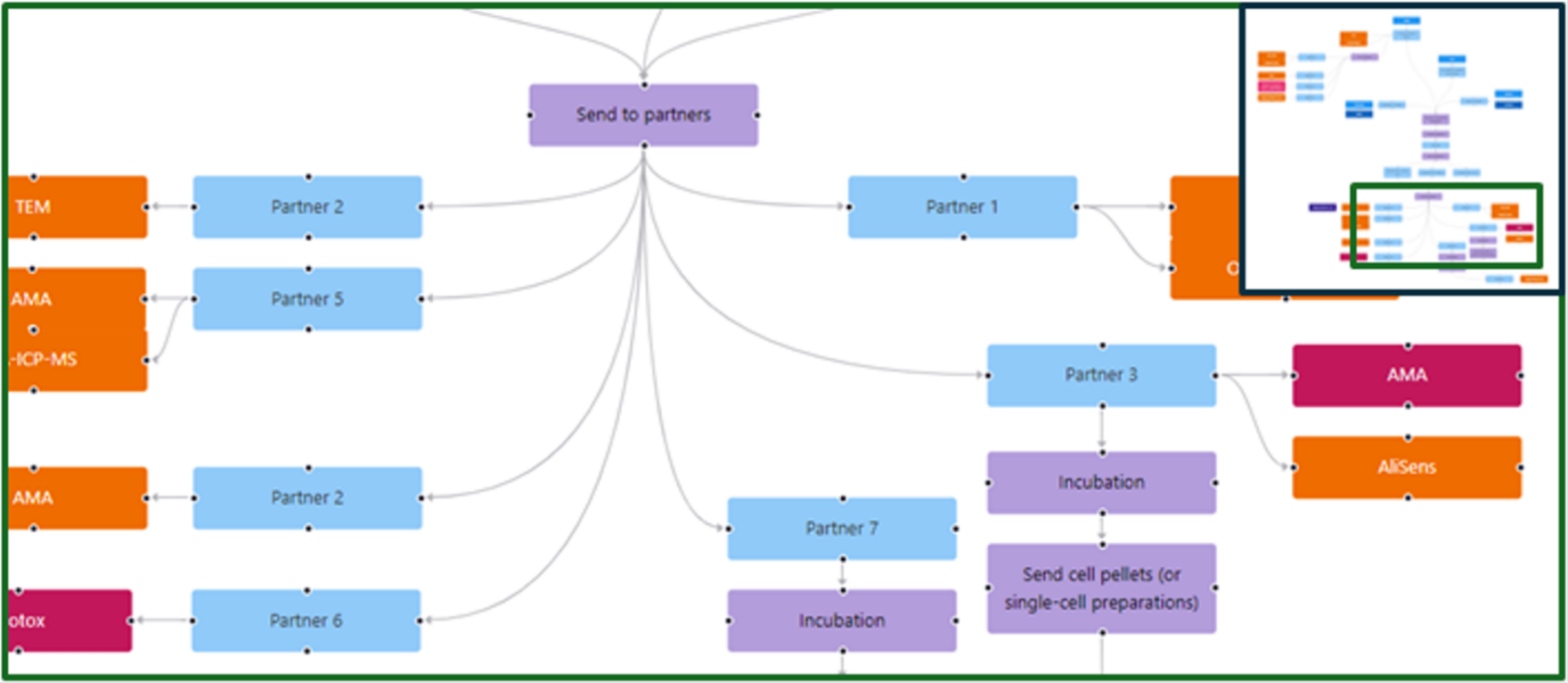
Part of the instance map for the MACRAMÉ use case of antibiotic-loaded nanoparticles representing the shipping of the pristine material to partners performing the human and ecotoxicity testing. The full instance map is available at https://figshare.com/articles/software/25416040?file=51103502 for interactive inspection.

Besides nodes representing the materials/samples (instances), characteristics and endpoints to be collected (experimentally and via text/database mining), and nodes describing modification steps applied to materials and samples (transformation protocols) and testing SOPs (sample preparation, measurement, and data processing), as described in the previous examples, instance maps also focus on and clearly define the chemical and physical treatment and processing steps performed as part of the manufacturing process, as well as shipping of samples from one partner to another (Figure 10).

By adding manufacturing and project management nodes, the instance maps now offer options to collect and document all digitalised information and results produced in upstream tasks of the case studies at one central place for direct (re)use in downstream tasks. In combination with the other components of the MACRAMÉ data management infrastructure and data harmonisation activities, the partners are able to adopt an on-the-fly FAIRification approach [31], in which all research output, including, but not limited to, sampling plans, study designs, in vitro and in silico method specifications, protocols, SOPs, and the data created, as well as guidelines, reports, training materials, and publications are directly shared – even in draft versions – by attaching them to instance maps nodes. The maps are only available to the consortium (until/unless they are made public by agreement of all involved parties). Hence, they can be used, for example, to report very detailed partly confidential information on the production processes needed for the life cycle assessment to evaluate energy and water consumption or as a basis to discuss the amounts of material needed to be shipped to the partners and then the status of the shipment. At the same time, the instance maps are continuously updated to increasingly represent the real workflows performed in the use cases with different versions, documenting the need and the reasons for deviating from the original planning and when this need became evident. We note here that instance maps could potentially also be utilised to pinpoint where a particular experiment has gone awry or deviated from prior results, including the case of negative data. This demonstrates that the FAIR concept is not only relevant for a secondary reuse of data. Instead, it also supports data collection and sharing in large projects from day 1, and the work invested in FAIRification at the planning stages will immensely reduce the effort for FAIR storage and sharing of data via agreed licences.

Finally, Figure 10 also demonstrates that misusing the colour coding established for clear identification of the node’s purpose can be beneficial during planning. Red colours, which are normally used to represent data, were here applied to indicate areas where further discussions were needed on how to perform the experiments or if they are even possible in the time frame and budget of the project. Extending the application of instance maps to all the uses described in this paper, and potentially many more, was only possible by not enforcing strict rules on how different node types can be connected. However, some more guidance might be needed to make the instance maps and the linked data more comparable and interoperable. Now that the applications are better defined and the use cases of the tool have matured, this will be pursued through extending the design guide published in the original instance map paper [28] and by preparing standardised workflows as arrangements of nodes and/or limiting the way nodes can be connected. To retain some flexibility, the node library could then be extended, for example, by adding specific planning nodes and/or by having customisable nodes.

### Key lessons from the implementation cases and future directions

Through the collaboration across the different implementation cases (presented here as examples 1–5) a number of additional features arose, which could extend the functionality of the InstanceMaps tool. To provide a concise, yet comprehensive, overview of the lessons learned, Table S1 in Supporting Information File 1 lists all data management features that were captured in each of the examples 1–5 above across the data management life cycle phases (collaboration planning, study design, study execution, data analysis and enrichment, and data validation and reuse, as defined in [29]), using an “X”. Some features that had to be applied manually and retrospectively are indicated as “(X)”. Requested features to be included in future updates of the currently available version are given in purple rows.

The modifications introduced during the development of the InstanceMaps tool, especially the extension of the available node types, opened up many new uses of instance maps for all of the applications presented above, and potentially new ones in the future. However, they also made defining rules on how to connect different nodes less straightforward than in the original approach, where the focus was completely on the fate of nanomaterials. Newer instance maps look into more detail of the biological testing system (see Figure 5). Transformation protocol nodes helped to understand which object (e.g., nanomaterial, biological test system, or solvent/medium) underwent a modification, but they made the separation between material and medium less obvious. These circumstances also raised the question as to whether materials and medium always have to be associated to an instance or if they can be independent entities when they are used for the first time in a synthesis, functionalisation, or exposure protocol (see Figure 4 and Figure 10).

Another example where different groupings of nodes have been used in different applications was the use of properties in combination with sample preparation, measurement, and processing protocols, as well as the resulting data. For example, the combination of sample preparation → measurement → raw data → processing → processed data, could be placed before or after the node defining the measured property, or could even replace this node completely. It was interesting to see how instance maps describing the same study but created by different users showed significant variations in how nodes were used and connected. This was first recognised when the study from Martinez et al. [32] was used in instance map training and then compared to the original map presented in the publication. Such deviations in instance map design do not cause a problem per se. In most cases, it was easy for others to understand the design and flow of the study and to easily identify important results based on common sense. Only in a few early cases, the maps needed to be corrected to avoid inconsistencies. The corresponding data, protocols/SOPs, and other research outputs could be linked to the maps independently of differences in representation.

To demonstrate that such variations in how instance maps are constructed and nodes are linked can be used to put the focus on different parts of the instance maps, we decided to present all of the examples in the way that the person(s) who performed the experiments had created the maps; we did not force users to comply with any specific set of rules. However, some more standardisation and a limited set of rules for linking nodes could speed up comparison (and interoperability) of workflows, one of the main benefits stated during the NIKC curation process. Standardisation would also facilitate the generation of harmonised, comparable data packages combining all information associated with one map, enabling upload of all data to target databases.

There are a number of other areas where the instance maps and the tool could be further extended. The highlighting of specific areas in the maps shown in Figure 3 and Figure 8 was created manually; but this clearly shows that integrating functionalities to create such annotations directly in the tool would be very beneficial. Additionally, better support to link different instance maps or to show more detail when hovering over specific parts could reduce the complexity of the maps, especially for complex studies as visualised in Figure 9 and Figure 10, without the need to remove important details. Finally, the data management and sharing functionality need to be improved to show which information is available and from where, to give access to multiple information sources from one node, and to provide integration with important data management tools such as ELNs and protocol repositories. Ways to implement these extensions and improvements are currently under investigation.

It is worth stressing, however, that even if instance maps could drastically change the way data is collected, they are not meant to replace existing data management solutions. Instead, tools implementing the instance map concept should be integrable into existing data ecosystems. Instance maps address two very specific purposes: (i) They provide a visual and structured overview of a study, and (ii) they are an addition to the original concept, linking to resources with additional information for a specific part or component of the study. In this way, they can become the link between different types of personal, institutional, and public information resources (databases and data warehouses, protocol and SOP repositories, software, and source code repositories) and data input and curation services including ELNs. Some ELNs already offer a somewhat similar functionality by allowing user to organise the different steps of the experiments in a workflow. However, as shown in this paper, the instance maps are one level above these workflows since they can represent different levels of detail to show complete, very complex studies, and then zoom into the details of these studies to highlight the metadata and data required at each step. Additionally, different solutions can be used for different types of information customised to the needs of the user and/or community recommendations and are not limited to what a specific ELN solution is offering.

## Conclusion

From its initial conception as a way to track nanomaterials’ transformations as reported in literature studies, the instance map approach has undergone very rapid development into a multipurpose experimental visualisation tool with multiple applications. At its simplest, an instance map can be considered as a graphical abstract summarising the steps in an experimental, computational, or combined workflow, demonstrating the materials, their surroundings (medium, environment, and organism), the endpoints being measured, and the data flows arising from each step of the experiment (synthesis, dispersion, characterisation, exposure, and hazard assessment) and/or each stage of the nanomaterials life cycle (production, formulation, application, end of life, and disposal or recycling). When applied to standardised regulatory tests or production scenarios, instance maps can be used to provide completeness checks for studies or production batches, ensuring that all necessary parameters to be recorded are captured in the visual model. In this context, instance maps can also be utilised as training tools to emphasise to researchers and operators why specific parameters or checks are essential and to ensure that the complete workflow is understood, even when individuals are only responsible for small segments of a workflow. Application of instance mapping at the study design stage can also provide critical insight into bottlenecks and support management aspects, such as flows of samples between partners in collaborative research projects and efforts to support FAIRification of metadata and data prior to data collection, to save time and resources later.

Creation of the instance mapping software tool described here has greatly enhanced the utility of instance maps, makes extended applications of instance mapping more accessible, and mapping of highly complex and/or multipartner collaborative workflows feasible and practical. The examples presented here highlight the flexibility of the instance mapping software tool, including the capacity for linking of instance maps, and for inclusion of additional category nodes covering quality assurance and quality control, industrial production, and management of (planned and actual) materials flows. This flexibility has allowed instance mapping to be used for designing experiments, developing SOPs, and creating and sharing workflows within projects, and as an additional data management tool. However, as the user base expands, the risk of emerging divergent approaches also increases, which will reduce its effectiveness for comparing and integrating datasets. Thus, a balance between flexibility and standardisation will be implemented, through guiding principles for the design of instance maps and the optimal connection of nodes, to maximise its potential for harmonisation and standardisation purposes. This would facilitate the generation of harmonised, comparable data packages combining all information associated with one map and enabling the upload of all data to target databases such as the NanoCommons Knowledge Base. Integration of the instance map tool with other data management solutions, such as electronic laboratory notebooks, protocols registries, and databases, will further enhance its utility and position it as a key FAIR-enabling resource for safety and sustainability assessment of nanoscale and advanced materials, and beyond.

## Supporting Information

All instance maps created in the new instance map tool are available from https://figshare.com/articles/software/25416040?file=51103502.

File 1 Overview of lessons learned by applying the InstanceMaps tool.

## Data Availability

The Instance Maps generated and analyzed during this study are openly available at https://figshare.com/articles/software/25416040?file=51103502.
