# Supplementary material for: Instance maps as an organising concept for complex experimental workflows as demonstrated for (nano)material safety research
Source: Beilstein J Nanotechnol. 2025 Jan 22;16:57–77. doi: 10.3762/bjnano.16.7 (PMC11773194; doi:10.3762/bjnano.16.7)
Supplement: File 1 — Overview of lessons learned by applying the InstanceMaps tool. [file Beilstein_J_Nanotechnol-16-57-s001.pdf]

BEILSTEIN JOURNAL OF NANOTECHNOLOGY

Supporting Information

for

Instance maps as an organising concept for complex experimental workflows as demonstrated for (nano)material safety research

Benjamin Punz, Maja Brajnik, Joh Dokler, Jaleesia D. Amos, Litty Johnson, Katie Reilly, Anastasios G. Papadiamantis, Amaia Green Etxabe, Lee Walker, Diego S. T. Martinez, Steffi Friedrichs, Klaus M. Weltring, Nazende Günday-Türeli, Claus Svendsen, Christine Ogilvie Hendren, Mark R. Wiesner, Martin Himly, Iseult Lynch and Thomas E. Exner

Beilstein J. Nanotechnol. **2025,** *16,* 57–77. doi:10.3762/bjnano.16.7

Overview of lessons learned by applying the InstanceMaps tool

License and Terms: This is a supporting information file under the terms of the Creative Commons Attribution License (https://creativecommons.org/licenses/by/4.0). Please note that the reuse, redistribution and reproduction in particular requires that the author(s) and source are credited and that individual graphics may be subject to special legal provisions.
The license is subject to the *Beilstein Journal of Nanotechnology* terms and conditions: (https://www.beilstein-journals.org/bjnano/terms)

Table S1: Overview of the lessons learned by applying the InstanceMaps tool to the five implementation cases (examples 1–5 in the main manuscript). Features applied within the five examples are indicated by “X”, while some features, indicated by “(X)” represent functionalities that had to be implemented manually. Features commonly agreed upon or requested for future updates of the InstanceMaps tool are listed in the bottom lines extending the use of the InstanceMaps tool even further, including embedding into data management systems.

Current and future Instance Map capacities group by data life cycle phases →

Examples provided ↓

	Examples provided	Collaboration Planning & Monitoring					Study Design					Execution			
		Multi-user functionality for collaborative creation of maps	Planning of partner involvement & responsibilities	Monitoring of workflow progress	Highlighting multidimensional relations / dependencies of process	Knowledge transfer	Easy overview of complex systems	Planning of data gap filling by testing and read-across	Attachment of pre-existing information	Planning of timing and relationship between experiments	Planning of (raw) material and sample flow	Attachment of resources without cluttering (metadata, protocols, different data types,...)	Documentation of material fate / material & data provence	Documentation of quality control measures	Documentation of handling of biological test systems
1	Documentation of nanomaterials synthesis and provision of unique identifiers for nanomaterials					X	X				X	X	X		
2	Monitoring nanomaterial transformation in complex environmental media					X	X	(X)				X	X		
3	Linking assay QA/QC with SOPs for running cultures of biological organisms and standardised ecotoxicity testing					X	X		X			X		X	X
4	Showcasing complex workflows in human immunotoxicity assessment using cell lines and primary cellular models				X	X	X			X	X	X	X	X	X
5	Using instance maps for planning and refining data and material workflows in large collaborative projects	X	X	X	X	X	X	X	X	X	X	X	X		
	Feature request for Instance Map Tool														
	Feature request for Data Management System														

	Examples provided	Data-Curation & -Enrichment										Data-Validation & -Re-Use		
		Visualisation of projects / studies / individual experimental workflows	Integration into existing data management workflows	On-the-fly data collection	Data FAIRification	Data capturing as structured scientific literature (i.e. data mining)	Data integration with data reporting standards	Data integration with electronic lab notebooks	Data integration with database solutions	Grouping of notes related to an experiment / part of study	Annotation of groups of nodes	Visual simplification (i.e. hiding of groups of nodes)	Visualisation of material-, sample- and data-provenance	Integration of regulatory requirements
1	Documentation of nanomaterials synthesis and provision of unique identifiers for nanomaterials	X			X	X						(X)	(X)	
2	Monitoring nanomaterial transformation in complex environmental media	X	X		X					(X)	(X)			
3	Linking assay QA/QC with SOPs for running cultures of biological organisms and standardised ecotoxicity testing	X	(X)	(X)	X		(X)	(X)	(X)					X
4	Showcasing complex workflows in human immunotoxicity assessment using cell lines and primary cellular models	X	(X)	(X)	X		(X)	(X)	(X)	(X)	(X)			X
5	Using instance maps for planning and refining data and material workflows in large collaborative projects	X	X	X	X		(X)	(X)	(X)					
	Feature request for Instance Map Tool									X	X	X	X	
	Feature request for Data Management System		X	X			X	X	X					
